# The usefulness of bioelectrical body composition analysis (BIA) in the proper assessment of nutritional status in children and adolescents with idiopathic scoliosis (IS)

**DOI:** 10.1186/1748-7161-8-S2-O35

**Published:** 2013-09-18

**Authors:** Jacek Durmala, Edyta Matusik, Jacek Durmala, Pawel Matusik, Karol Wadolowski

**Affiliations:** 1 Department of Rehabilitation, Medical University of Silesia, Katowice, Poland; 2 Department of Pediatrics, Pediatric Endocrinology and Diabetes, Medical University of Silesia, Katowice, Poland

## Background

Based on our recent data, nutritional status disturbances (both under- and overweight) can be associated with the severity of scoliotic curve. 

## Purpose

The study objective was to compare two methods for the assessment of nutritional status (BMI vs. BIA-body composition analysis by bioelectrical impedance analyzer) in IS patients. 

## Methods

For a total of 317 IS patients (240 girls and 77 boys), mean age 14.11±2.79y, the scoliotic curve was assessed by Cobbs angle and angle vertebra rotation (AVR). Height, weight, waist and hip circumferences were measured and BMI, BMI Z-score, waist/height ratio (WHtR) and waist/hip ratio (WHR) were calculated for the entire group. Body composition parameters, such as fat mass (FAT), fat-free mass (FMM), predicted muscle mass (PMM) and total body water (TBW), were evaluated using a bioelectrical impedance analyzer. Nutritional status was classified by centile charts for BMI as underweight, normal weight, overweight or obese, and for FAT% as underfat, lean, overfat or adiposity. This was a prospective, randomized study. 

## Results

Nutritional status assessed by BMI has been associated with 21.1% of misclassification compared to BIA. There were important differences between the percentage of underweight vs. underfat patients (13.9% vs. 9.5%), overweight vs. overfat (5.4% vs. 7.9%) and obesity vs. adiposity (2.8% vs. 5.0%). There was no significant correlation between BMI and scoliosis severity in the subgroups classified by standard measurement. However, the BMI Z-score correlated significantly with Cobb and AVR in every BMI-classified subgroup. There were also significant correlations between body composition parameters (BIA) and vertebral deformity in only the normal BMI group. After the correction to the FAT%, 252 (78.9%) children were properly classified; of this group of IS patients, statistical analysis showed strong (p<0.001), significant correlation between either Cobb’s angle or AVR vs. every (standard and bioelectrical) anthropometrical parameter. 

## Conclusions and discussion

Nutritional status classification by BMI assessment overestimates the underweight and leads to the underestimation of both overweight and obese patients with IS. Bioelectrical impedance analysis is a useful tool for the proper nutritional status assessment in the pediatric population with IS. Properly assessed nutritional status is significantly associated with the severity of scoliotic curve assessed by Cobb’s angle and AVR. 
